# Elucidating the Material Basis and Receptor Mechanism of Bitterness in *Castanopsis fissa* Honey Using Machine Learning, Metabolomics, and Molecular Docking

**DOI:** 10.3390/foods15081379

**Published:** 2026-04-15

**Authors:** Yaxi Zhou, Dong Xu, Meichao Bu, Fei Pan, Hualei Chen, Wenjun Peng, Wenli Tian

**Affiliations:** State Key Laboratory of Resource Insects, Institute of Apicultural Research, Chinese Academy of Agricultural Sciences, Beijing 100093, China; 15239407080@163.com (Y.Z.); 82101235554@caas.cn (D.X.); bmc18726625871@163.com (M.B.); yunitcon@yeah.net (F.P.); chenhualei@caas.cn (H.C.)

**Keywords:** alkaloids, TAS2R46, electronic tongue, UPLC-QQQ-MS/MS, taste markers

## Abstract

The distinctive bitter profile of *Castanopsis fissa* honey (LSZH) has not yet been clearly characterized at the chemical and molecular levels. Based on the LSZH samples (*n* = 6), this study investigated bitterness-associated compounds and their potential receptor interactions by integrating sensory evaluation, machine learning, untargeted metabolomics, electronic tongue analysis, targeted UPLC-QQQ-MS/MS quantification, and molecular docking. A Random Forest model combined with untargeted metabolomics screened 71 candidate bitter compounds, among which alkaloid-related metabolites were prominently represented. Electronic tongue analysis showed that several compounds exhibited higher bitterness-related sensor responses than quinine under the tested conditions. Targeted UPLC-QQQ-MS/MS analysis identified and quantified five key compounds, among which kynurenic acid was the most abundant, reaching approximately 4500 ppm (mg/kg). Molecular docking suggested that these compounds could favorably interact with the human bitter taste receptor TAS2R46, with binding affinities ranging from −5.4 to −6.5 kcal/mol, mainly through hydrogen bonding, hydrophobic interactions, and π-related interactions. Overall, this study provides chemical evidence and mechanistic clues for understanding the bitterness of LSZH and offers an integrated analytical framework for screening bitterness-associated compounds in complex food systems.

## 1. Introduction

Honey, characterized by its abundant nutritional value and unique flavor profile, is widely consumed worldwide. Its flavor is determined not only by the sweetness imparted by sugars but is also closely associated with the secondary metabolites derived from botanical sources [1]. *Castanopsis fissa* honey (LSZH), predominantly produced in southern China, is a highly distinctive regional monofloral honey [2]. Unlike common varieties such as acacia or lychee honey, which are characterized by a clear sweetness, LSZH exhibits pronounced bitterness and astringency in sensory evaluations. In food science, bitterness is typically associated with plant-derived substances such as alkaloids, polyphenols, or glycosides [3]. However, the chemical basis responsible for the characteristic bitterness of LSZH remains unclear. In addition, previous studies have shown that bitterness in honey and other plant-derived foods is often associated with specific classes of secondary metabolites, including alkaloids, phenolic compounds, and glycosides [4]. Alkaloid-related compounds are widely recognized as important contributors to bitterness due to their strong interaction with human bitter taste receptors (TAS2Rs) [5]. Similarly, phenolic compounds such as flavonoids and phenolic acids have also been reported to contribute to bitter and astringent taste attributes in various food systems [6]. Although these compound classes have been identified in different natural products and, to some extent, in honey matrices, the specific chemical basis underlying the characteristic bitterness of LSZH and its potential receptor interaction mechanisms remain insufficiently understood. Therefore, a systematic investigation integrating chemical profiling and mechanistic analysis is needed to clarify the origin of bitterness in this distinctive honey.

The traditional discovery of food flavor compounds predominantly relies on sensory-guided fractionation coupled with gas or liquid chromatography-mass spectrometry. However, given that honey is an extremely complex matrix containing thousands of metabolites, conventional approaches are not only time-consuming and labor-intensive but also struggle to efficiently pinpoint target taste molecules within vast untargeted metabolomics datasets. In recent years, alongside the rapid advancement of high-resolution mass spectrometry, accurately screening compounds with specific sensory attributes from an overwhelming array of metabolites has emerged as a major challenge in food flavor chemistry [7,8].

Machine learning (ML), serving as a robust computational screening tool, offers a novel perspective to overcome this bottleneck. By utilizing established databases such as BitterDB [9] and FlavorDB [10], substantial datasets can be acquired for model training. ML algorithms, including Random Forest, XGBoost, and LightGBM, can deeply learn the nonlinear mapping relationships between molecular structural features and taste attributes, thereby enabling high-throughput and high-precision classification predictions for unknown metabolites [11,12]. Integrating ML with untargeted metabolomics can dramatically narrow down the screening scope for targeted taste-active substances, enhancing the efficiency and accuracy of identifying characteristic flavor compounds in complex systems [13,14].

Furthermore, the ultimate manifestation of flavor perception relies on the specific binding between ligand molecules and human taste receptors. The human perception of bitterness is mediated by a family of G protein-coupled receptors (TAS2Rs) located on taste buds [15]. Following the recent elucidation of the high-resolution three-dimensional structure of the crucial human bitter taste receptor TAS2R46 [16], molecular docking technology has been widely adopted for the discovery and validation of bitter compounds in food [17]. TAS2R46 was selected as a representative receptor for molecular docking because its high-resolution structure has been resolved and it is known to respond to a broad range of bitter compounds. At the atomic scale, molecular docking can simulate the binding conformations of screened bitter small molecules with target receptor proteins and reveal their potential interactive forces, thereby providing robust computational validation for the physiological mechanisms underlying the bitterness of these compounds [18]. Bitterness perception is mediated by the interaction between small molecules and human bitter taste receptors (TAS2Rs), which serve as the molecular interface linking chemical stimuli to sensory perception. Therefore, identifying bitterness-associated compounds and evaluating their potential interactions with TAS2Rs provide a conceptual bridge between chemical composition and perceived bitterness.

It should be noted that machine learning predictions are dependent on the quality and distribution of the training data and may be subject to bias. Similarly, molecular docking provides theoretical insights into potential ligand–receptor interactions, but does not directly confirm receptor activation or biological activity.

To address these challenges, this study proposes an integrated and efficient analytical framework for the systematic identification of bitter compounds in complex food matrices. Compared with traditional sensory-guided fractionation approaches, our strategy combines machine learning-assisted prediction with untargeted metabolomics to enable high-throughput and targeted screening of candidate bitter molecules. Specifically, a Random Forest model trained on public taste databases was employed to establish structure–taste relationships and prioritize potential bitter compounds from large-scale metabolomics data. Subsequently, a multi-step validation workflow was implemented, including sensory evaluation to confirm the bitter phenotype of LSZH, electronic tongue analysis for rapid taste verification, targeted UPLC-QQQ-MS/MS for absolute quantification of key compounds, and molecular docking to explore ligand–receptor interactions at the molecular level. By integrating computational prediction with experimental validation, this workflow not only improves screening efficiency but also provides mechanistic insights into the interaction between bitter compounds and human taste receptors. Based on this, we hypothesize that specific metabolites, particularly alkaloid-related compounds, contribute to the characteristic bitterness of LSZH and may interact with human bitter taste receptors.

## 2. Materials and Methods

### 2.1. Materials

*Castanopsis fissa* honey (LSZH) samples (*n* = 6) were collected in 2023 from multiple apiaries in Cangyuan County, Yunnan Province, China (98°52′–99°43′ E, 23°04′–23°40′ N). The samples were obtained from different hives. Acacia honey (*Robinia pseudoacacia*, YHH), lychee honey (*Litchi chinensis*, LZH), and dandelion honey (PGYH) were collected in 2023 from local apiaries in China.

Chemical standards including kynurenic acid, 8-hydroxyquinoline, 2,4-quinolinediol, erucamide, adenine, xanthurenic acid, oleamide, 4-indolecarbaldehyde, harmaline, uridine, 2-hydroxyquinoline, and linoleic acid were purchased from Shanghai Yuanye Bio-Technology Co., Ltd. (Shanghai, China). The standard *p*-cymene was obtained from Shanghai Aladdin Biochemical Technology Co., Ltd. (Shanghai, China). All other reagents used were of analytical grade.

### 2.2. Sensory Evaluation

The sensory evaluation for this study was conducted in a standardized sensory laboratory. According to the GB/T 16291.1-2012 standard [19], a sensory panel of 10 trained evaluators (5 males and 5 females, aged 23–28 years) was selected. The sweetness, bitterness, astringency, aroma, and overall flavor of LSZH, YHH, LZH, and PGYH were evaluated using a 5-point intensity scale ranging from 1 to 5, where “1” indicated none and “5” indicated the strongest intensity. The 5-point scale was adopted to improve scoring consistency and reduce variability among trained panelists, particularly for distinguishing key taste attributes such as bitterness and astringency [20]. All participants were informed about the study and provided written informed consent prior to participation.

### 2.3. Machine Learning

Molecules annotated as bitter, tasteless, and sweet were collected from public taste databases and used to construct the machine learning dataset. In total, the dataset comprised 1772 bitter molecules, 196 tasteless molecules, and 1229 sweet molecules, which were labeled as 0, 1, and 2, respectively [21]. To numerically represent molecular structural features, the mol2vec algorithm was used to generate 300-dimensional molecular embeddings for each compound [22]. Four commonly used machine learning algorithms, namely XGBoost, LightGBM, Random Forest, and multilayer perceptron (MLP), were employed for model construction. The dataset was randomly divided into a training set and an independent validation set at a ratio of 8:2. During model training, 10-fold cross-validation was performed on the training set to reduce overfitting and improve model robustness. In each fold, nine subsets were used for training and the remaining subset was used for internal validation.

To improve model performance, hyperparameter optimization was performed for each algorithm, and the optimal parameter combinations were selected based on cross-validation performance. After training, the final models were further evaluated using the independent validation set. Considering the moderate predictive performance of the models, the machine learning analysis in this study was used as a screening strategy to prioritize candidate bitter compounds from untargeted metabolomics data, rather than as definitive evidence of bitterness.

### 2.4. Model Evaluation

Model performance was evaluated using confusion matrices generated by the confusion_matrix function in the sklearn.metrics module [23]. Based on the confusion matrix, the numbers of true positives (TPs), false positives (FPs), false negatives (FNs), and true negatives (TNs) were obtained for each model. Four commonly used evaluation metrics, including Accuracy (ACC), Precision, Recall, and F1-score (F1), were calculated according to Equations (1)–(4), respectively. These metrics were used to comprehensively assess classification performance from different aspects, including overall accuracy, positive prediction reliability, sensitivity, and the balance between precision and recall.

(1) $$ACC=\frac{TP+TN}{TP+FP+FN+TN}$$

(2) $$Precision=\frac{TP}{TP+FP}$$

(3) $$F1=\frac{2TP}{2TP+FP+FN}$$

(4) $$Recall=\frac{TP}{TP+FN}$$

### 2.5. Untargeted Metabolomics of LSZH

Untargeted metabolomics analysis of the LSZH samples was performed using solid-phase extraction (SPE) combined with ultra-high-performance liquid chromatography-high-resolution mass spectrometry (UPLC-HRMS). A Bond Elut PPL SPE cartridge (1225001) was sequentially conditioned and equilibrated with methanol (10 mL) and ultrapure water (10 mL). Exactly 5 g of LSZH was weighed and dissolved in 10 mL of ultrapure water. The mixture was vortexed and centrifuged, and the supernatant was collected as the honey solution. The solution was loaded onto the cartridge, washed with ultrapure water (10 mL), and the target analytes were eluted with methanol (7 mL). The eluate was dried under a gentle stream of nitrogen and stored at −20 °C for future use. This procedure was repeated in triplicate.

The analysis was carried out using a UPLC system coupled with a Q Exactive Plus mass spectrometer (Thermo Fisher Scientific, Waltham, MA, USA). Chromatographic separation was achieved on a ZORBAX RRHD Eclipse Plus C18 column (3.0 × 150 mm, 1.8 μm) with a column temperature of 40 °C and an injection volume of 2 μL. Mobile phase A consisted of ultrapure water (LC–MS grade) containing 0.1% (*v*/*v*) formic acid (analytical grade), and mobile phase B consisted of methanol (LC–MS grade) containing 0.1% (*v*/*v*) formic acid. The gradient elution program was set as follows: 0–2 min, 95% A; 2–20 min, A linearly decreased from 95% to 0%; 20–30 min, 0% A. The mass spectrometer was equipped with an electrospray ionization (ESI) source. The spray voltages for positive and negative ion modes were set at 3500 V and 3000 V, respectively. The capillary temperature was 320 °C, the sheath gas flow rate was 40 arb, and the scan range was *m*/*z* 80–1200 with a full MS resolution of 17,500. The collision energies were set at 20, 30, and 40 eV. Full MS scans were used for quantitative analysis, while data-dependent MS/MS scans (Full MS/dd-MS^2^) were employed for qualitative analysis. Data processing was performed using Xcalibur 3.1 software.

### 2.6. Metabolite Analysis and Bitterness Prediction

Bitter metabolites in honey were identified based on metabolomics data and matched with data from *Apis cerana* honey. NPClassifier was then used to annotate the metabolite categories [24], and the results were visualized based on metabolite classes and relative contents. The bitter, tasteless, and sweet prediction algorithms were applied to predict the tastes of the honey metabolites, leading to the identification of 71 bitter substances, 43 sweet substances, and 3 tasteless substances. The top 20 bitter metabolites were selected based on their relative abundance in LSZH, combined with the bitterness prediction results from the machine learning model. Compounds that were both predicted as bitter and exhibited relatively high abundance were prioritized for subsequent experimental validation.

### 2.7. Electronic Tongue

The ASTREE-II electronic tongue system (Alpha MOS, Toulouse, France) was employed in this experiment. The system is equipped with seven sensors, including a sourness sensor (AHS), general purpose sensor (PKS), saltiness sensor (CTS), umami sensor (NMS), general purpose sensor (CPS), sweetness sensor (ANS), and bitterness sensor (SCS), to analyze the taste profiles of the standards. For sample preparation, standard compounds were formulated into 10% ethanol solutions at a concentration of 0.1 mg/mL. During detection, the sample solutions were poured into specialized sample cups. The sensor cleaning time was set to 5 min, the sample detection time to 30 s, and the aftertaste measurement time to 30 s. Each sample was tested in 10 parallel replicates. After the detection, data were acquired using the software provided with the electronic tongue. The sensor response values were first normalized using min–max scaling to a range of [0, 1] according to Equation (5). Subsequently, quinine was used as a reference standard, and the normalized bitterness response of each compound was compared relative to that of quinine to allow cross-sample comparison.

(5) $$X_{norm}=\frac{X-X_{min}}{X_{max}-X_{min}}$$

X: original data; Xmin: minimum value of the data; Xmax: maximum value of the data; Xnorm: normalized data.

### 2.8. UPLC-QQQ-MS/MS Validation

An Agilent UPLC-QQQ-MS/MS system (Agilent Technologies, Santa Clara, CA, USA) was used to determine the content of bitter compounds in the honey. Mobile phase A consisted of ultrapure water containing 0.1% (*v*/*v*) formic acid, and mobile phase B was methanol. The flow rate was set to 0.3 mL/min with an injection volume of 2 μL. The column temperature was maintained at 40 °C. The instrument configuration included a multisampler, binary pump, column compartment, variable wavelength detector (VWD), and a triple quadrupole (QQQ). The ion source was AJS ESI. The stop time was 1 min, and the VWD scan range was from 190 nm to 400 nm. The gas temperature was 290 °C, the gas flow was 11 L/min, and the nebulizer pressure was 45 psi.

### 2.9. Molecular Docking

The 3D structures of the bitter molecules (kynurenic acid, 8-hydroxyquinoline, 2,4-quinolinediol, adenosine, uridine, and quinine) were downloaded from the PubChem database and converted into PDB format using the PyMOL program (Version 3.10). Subsequently, Avogadro software was used to perform geometric optimization on bitter molecules based on the MMFF94s force field to eliminate unreasonable bond lengths and angles [25].

The PDB structure of the bitter taste receptor (TAS2R46) (PDB ID: 7XP4) was retrieved and downloaded from RCSB PDB. Based on previous research [16], the GetBox plugin was used to define the docking grid box for TAS2R46 (center_x = 147.1, center_y = 146.1, center_z = 112.0; size_x = 40, size_y = 40, size_z = 40). Finally, following previously reported protocols, AutoDock Tools (Version 1.5.7) was utilized to prepare the structures of the bitter molecules and the receptor by sequentially adding hydrogens, calculating Gasteiger charges, and merging non-polar hydrogens [26]. Semi-flexible molecular docking was then performed using the AutoDock Vina program [27]. After docking, the PyMOL program and Discovery Studio 4.5 were used for visualization and interaction analysis.

### 2.10. Statistical Analysis

Data were expressed as the mean ± standard deviation (SD). Raw UPLC-QE-MS data were processed using Compound Discoverer 3.3.1.111 (Thermo Fisher Scientific, Waltham, MA, USA), including peak detection, retention time alignment, and compound extraction using the default workflow. Data normalization was performed to reduce analytical variability across samples. Metabolite annotation was conducted based on accurate mass and MS/MS fragmentation matching against the mzCloud database. Only metabolites with reliable spectral matching were retained for subsequent analysis. The UPLC-QQQ-MS/MS data were analyzed quantitatively using Agilent MassHunter software (Version 12.0).

## 3. Results and Discussion

### 3.1. Sensory Evaluation Analysis

**Figure 1:**
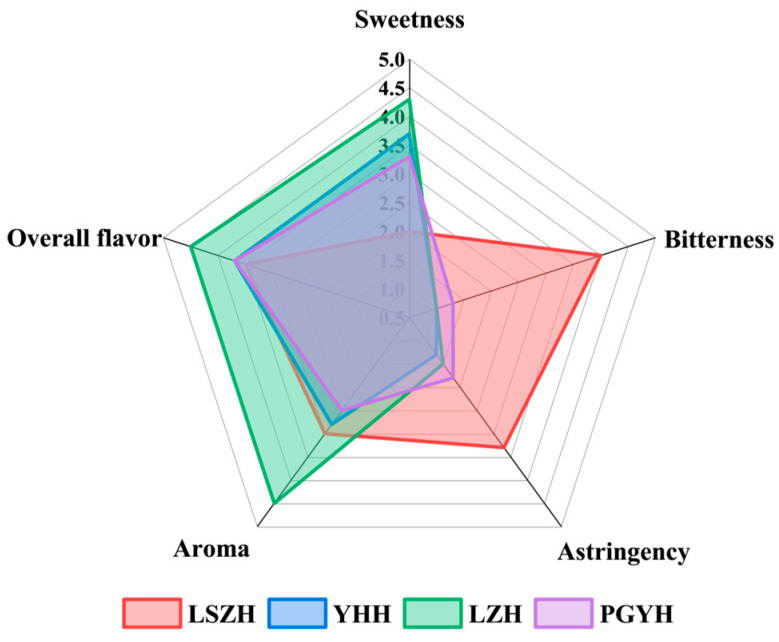
Results of the sensory evaluation analysis.

The sensory evaluation of the four types of honey was conducted in a standard sensory laboratory. By scoring the sweetness, bitterness, astringency, aroma, and overall flavor of the honeys, we demonstrated the sensory differences between LSZH and the other honeys. The results are shown in Figure 1. As illustrated in Figure 1, LSZH exhibited significant bitterness and astringency, whereas the other three honeys showed almost no bitterness or astringency. In contrast, the sweetness and aroma of YHH, LZH, and PGYH were more prominent, distinctly setting them apart from LSZH. In terms of overall flavor, LZH was the most preferred, while no significant differences were observed among the other honeys. These results indicate that LSZH possesses a relatively distinct bitter taste that significantly differentiates it from other honeys, which highlights its unique characteristic.

### 3.2. Construction and Evaluation of Machine Learning Models

**Figure 2:**
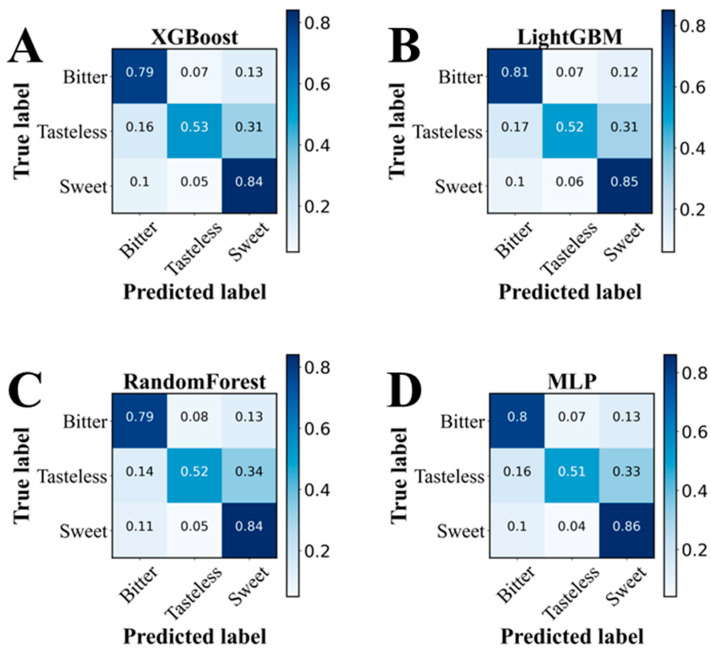
Confusion matrices of the four machine learning algorithms in the training set. (**A**) XGBoost; (**B**) LightGBM; (**C**) RandomForst; (**D**) MLP.

**Figure 3:**
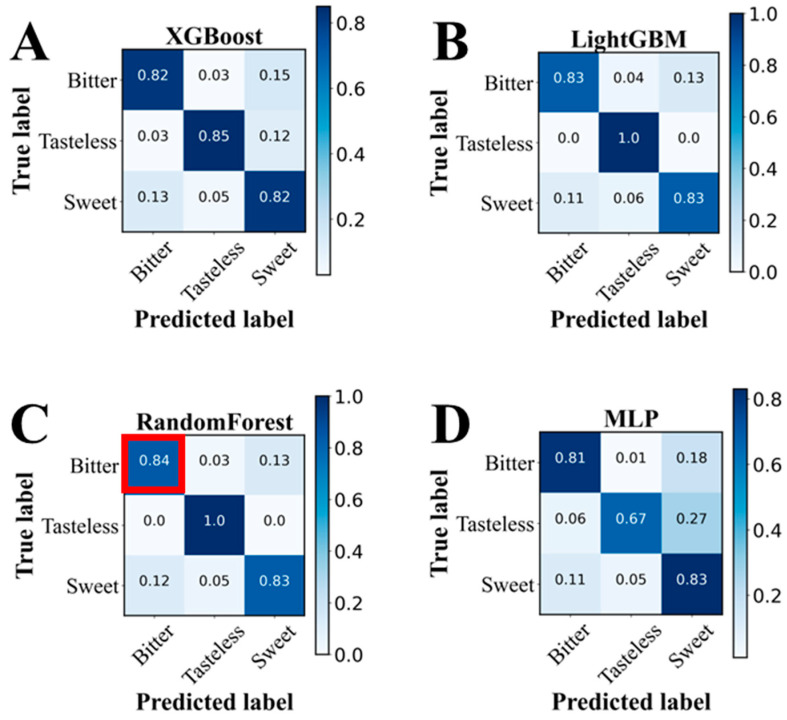
Confusion matrices of the four machine learning algorithms in the independent test set. (**A**) XGBoost; (**B**) LightGBM; (**C**) RandomForst; (**D**) MLP.

During model construction, the confusion matrices for four machine learning algorithms, XGBoost, LightGBM, Random Forest, and MLP, were obtained. Figure 2 presents the confusion matrices for the training set. As shown in the figure, the prediction accuracies of XGBoost, LightGBM, Random Forest, and MLP for bitter molecules are 0.79, 0.81, 0.79, and 0.80, respectively. The four machine learning algorithms showed moderate performance in distinguishing bitter compounds from tasteless and sweet compounds, indicating their utility as screening tools for candidate bitter metabolites. Figure 3 displays the confusion matrices for the independent test set, revealing that the prediction accuracies of XGBoost, LightGBM, Random Forest, and MLP for bitter molecules are 0.82, 0.83, 0.84, and 0.81, respectively. The Random Forest algorithm exhibited the highest prediction accuracy. Among the tested models, Random Forest showed relatively better predictive performance on the independent validation set and was therefore selected as the screening model for subsequent analysis (Table S1).

In fact, the results of our model construction are consistent with previous studies, which have also utilized the Random Forest algorithm to predict the bitterness of compounds. For instance, Bitter-RF, a highly efficient identification tool for bitter peptides, also employs the Random Forest algorithm and achieves a 98% accuracy in recognizing bitter peptides [28]. Similarly, BitterSweetForest is a Random Forest-based binary classifier utilized to predict the bitterness and sweetness of chemical compounds [29]. These findings indicate that the machine learning model adopted in our study is highly feasible for predicting bitter molecules in honey.

### 3.3. Untargeted Metabolomics Analysis of LSZH and Identification of Bitter Molecules

**Figure 4:**
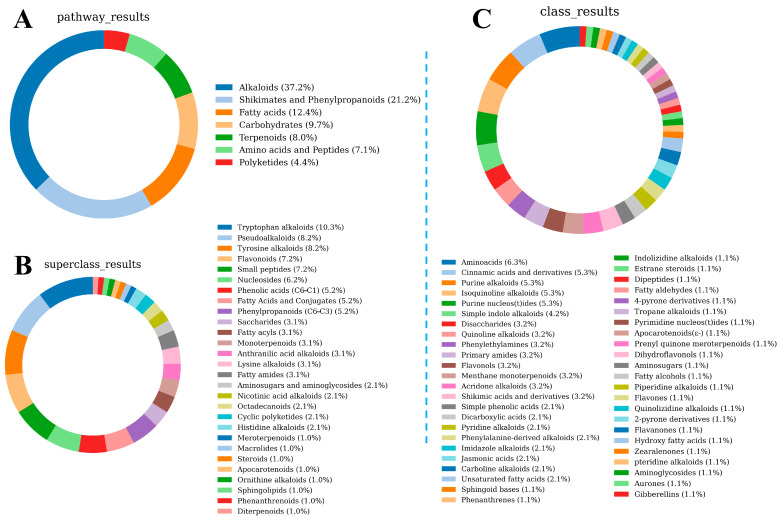
Class distribution of metabolites in LSZH. (**A**) Donut chart based on the biogenetic pathways of the compounds. (**B**) Donut chart classified by Superclasses. (**C**) Donut chart classified by Classes.

Based on the metabolomics data, we identified 10,732 compounds in LSZH and annotated their metabolite classes using NPClassifier. Figure 4 illustrates the class distribution of the different compounds in LSZH. Figure 4A is a donut chart based on the biogenetic pathways of the compounds, encompassing seven major categories: Alkaloids, Shikimates and Phenylpropanoids, Fatty acids, Carbohydra tes, Terpenoids, Amino acids and Peptides, and Polyketides. As shown in Figure 4A, Alkaloids exhibited the highest diversity in LSZH, accounting for 37.2% of all compounds, followed by Shikimates and Phenylpropanoids (21.2%), Fatty acids (12.4%), Carbohydrates (9.7%), Terpenoids (8.0%), Amino acids and Peptides (7.1%), and Polyketides (4.4%). Figure 4B is a donut chart classified by Superclasses, where the primary compound categories are Tryptophan alkaloids (10.3%), Pseudoalkaloids (8.2%), Tyrosine alkaloids (8.2%), Flavonoids (7.2%), Small peptides (7.2%), and Nucleosides (6.2%). Figure 4C is a donut chart classified by Classes, with the primary categories being Amino acids (6.3%), Cinnamic acids and derivatives (5.3%), Purine alkaloids (5.3%), Isoquinoline alkaloids (5.3%), Purine nucleos(t)ides (5.3%), and Simple indole alkaloids (4.2%). These results indicate that, whether classified by Classes or Superclasses, the most predominant metabolite types in LSZH originate from Alkaloids, especially Tryptophan alkaloids, which account for 10.3% of all metabolite types.

The dominance of alkaloid-related metabolites observed in LSZH is consistent with previous studies reporting that alkaloids are major contributors to bitterness in plant-derived foods [30]. Alkaloids are known to interact strongly with human bitter taste receptors (TAS2Rs), which may explain the pronounced bitter perception of LSZH. In addition, the high diversity of alkaloid subclasses, such as tryptophan and isoquinoline alkaloids, suggests that multiple structurally distinct compounds may contribute synergistically to the overall bitter profile.

**Figure 5:**
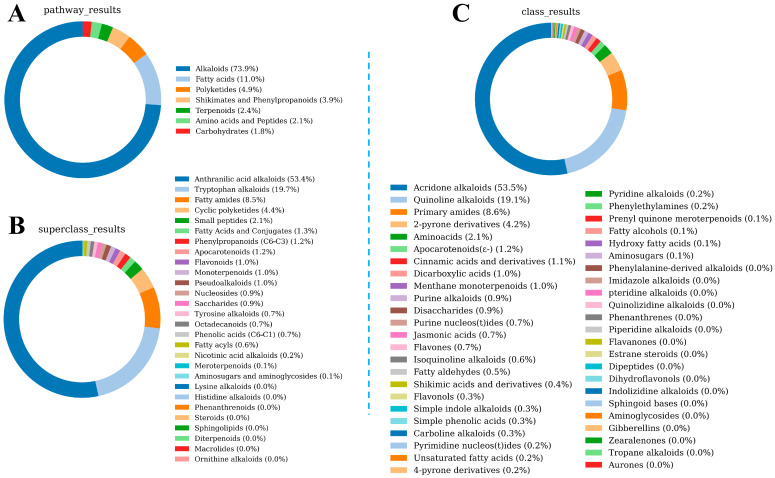
Donut charts based on compound contents and classifications. (**A**) Donut chart based on biogenetic pathways and contents. (**B**) Donut chart of contents classified by Superclasses. (**C**) Donut chart of contents classified by Classes.

The taste presentation of compounds in LSZH depends not only on the types of metabolites but also on the relative contents of specific components. Taking bitterness as an example, assuming a constant bitterness threshold, a higher content of bitter compounds increases the likelihood that the food will present a bitter taste. Therefore, we further plotted a donut chart of the metabolites in LSZH based on compound classes and their relative contents (Figure 5). Figure 5A shows the distribution by biogenetic pathway and content; the relative contents of the seven major categories—Alkaloids, Shikimates and Phenylpropanoids, Fatty acids, Carbohydrates, Terpenoids, Amino acids and Peptides, and Polyketides—are 73.9%, 11.0%, 4.9%, 3.9%, 2.4%, 2.1%, and 1.8%, respectively. Figure 5B displays the content distribution by Superclasses, with higher contents observed for Anthranilic acid alkaloids (53.4%), Tryptophan alkaloids (19.7%), Fatty amides (8.5%), and Cyclic polyketides (4.4%). Figure 5C shows the content distribution by Classes, with high contents of Acridone alkaloids (53.5%), Quinoline alkaloids (19.1%), Primary amides (8.6%), and 2-pyrone derivatives (4.2%). The content-based distribution further highlights that alkaloids not only exhibit high diversity but also dominate in relative abundance. Considering that taste perception is influenced by both compound concentration and sensory thresholds, the high abundance of these alkaloid-related metabolites suggests their potential relevance to the bitterness of LSZH. However, their actual contribution to bitterness requires further validation, as high abundance alone does not necessarily indicate sensory activity.

Previous studies have shown a very close relationship between Alkaloids and bitterness; that is, Alkaloids are considered one of the most important natural substances contributing to “bitterness”. For instance, the bitterness of faba beans originates from their Alkaloids and saponins [5]. The bitter taste of lupin seeds is also attributed to Alkaloids and is positively correlated with the Alkaloid content [31]. The results of this study revealed a high content and wide variety of Alkaloids in LSZH, which is likely the direct cause of its bitter taste.

**Figure 6:**
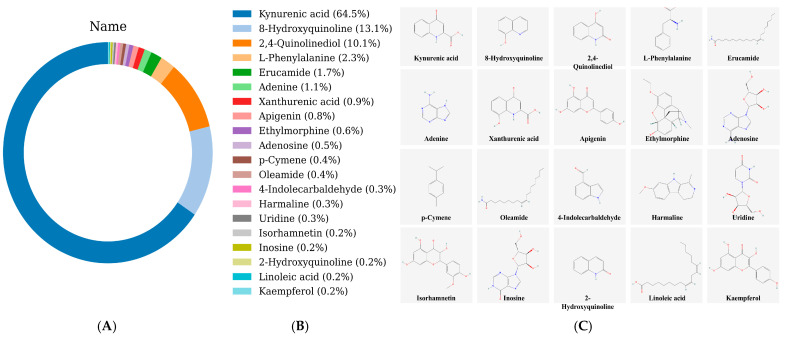
Class and content distribution of the 71 bitter compounds. (**A**) Donut chart based on biogenetic pathways and contents. (**B**) Donut chart of contents classified by Superclasses. (**C**) Donut chart of contents classified by Classes.

Furthermore, the bitter, tasteless, and sweet prediction algorithms were used to predict the LSZH metabolites, identifying a total of 71 bitter substances, 43 sweet substances, and 3 tasteless substances. We visualized the class and content distribution of the 71 bitter substances, as shown in Figure 6. Figure 6A demonstrates that the 71 predicted bitter substances are distributed across seven pathways, with the alkaloid pathway accounting for 92% of the total bitter substance content. The Superclass results revealed that over 65.7% of the bitter substance content originates from Anthranilic acid alkaloids. The Class results showed that 65.7% of the bitter substance content comes from Acridone alkaloids. These findings mutually corroborate the previous results, proving that the primary source of bitterness in LSZH is Alkaloids.

**Figure 7:**
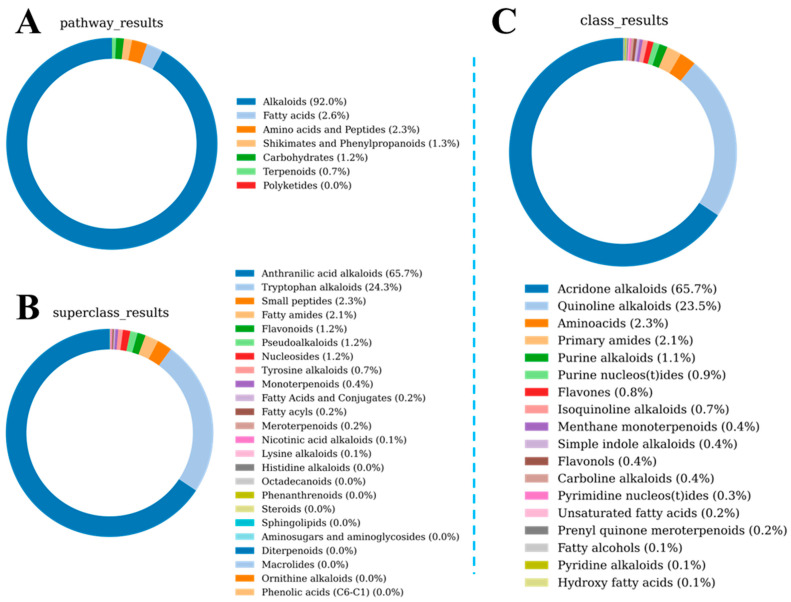
The top 20 bitter metabolites in LSZH based on relative content.

Figure 7 displays the top 20 bitter metabolites in LSZH based on relative content. It can be seen that kynurenic acid has the highest content (64.5%), followed by 8-hydroxyquinoline (13.1%) and 2,4-quinolinediol (10.1%), all exceeding 10%. This suggests that these compounds are candidate contributors to the bitter profile of LSZH based on their relative abundance, although their actual sensory contribution requires further validation.

### 3.4. Electronic Tongue Analysis

**Figure 8:**
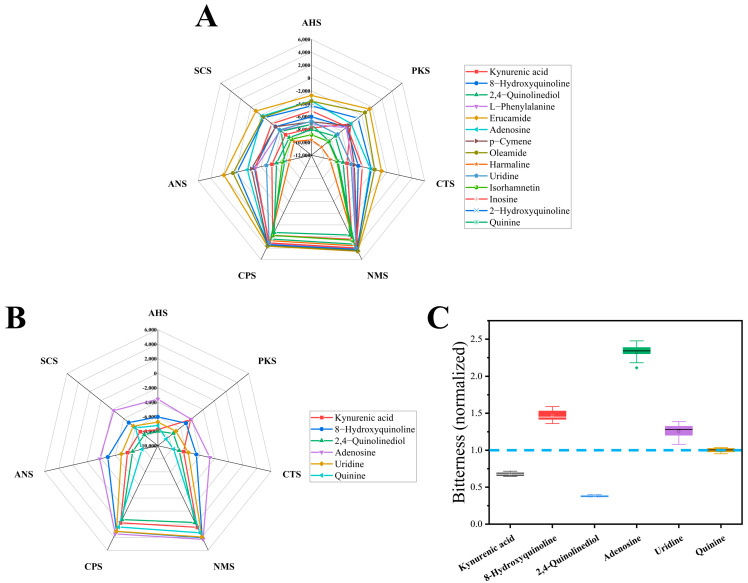
Electronic tongue analysis of bitter compounds in LSZH. (**A**) Taste radar chart of bitter compound standards. (**B**) Taste radar chart of the five identified bitter compounds in LSZH. (**C**) Normalized bitterness response values of the bitter compounds.

To investigate whether the compounds predicted by machine learning actually possess bitterness, we purchased chemical standards and performed electronic tongue testing, comparing them with quinine, a known bitter standard [32]. Among the top 20 compounds predicted to be bitter based on content, six have already been reported to have a bitter taste: L-phenylalanine [33], apigenin [34], adenosine [35], isorhamnetin [34], inosine [35], and kaempferol [34]. To verify that our predicted compounds indeed exhibit bitterness, we purchased the available standards for 13 compounds, conducted electronic tongue analysis, and compared them with quinine. The results are shown in Figure 8A, which is the taste radar chart of the bitter compound standards. It shows that each compound has a different response value to the various sensors. Notably, based on the response values of the bitterness sensor (SCS), except for kynurenic acid, 2,4-quinolinediol, isorhamnetin, and harmaline, the bitterness response values of the other compounds were all higher than that of quinine, indicating that these compounds elicit a higher bitterness-related sensor response than quinine.

### 3.5. Quantitative Analysis of Bitter Compounds in LSZH

**Figure 9:**
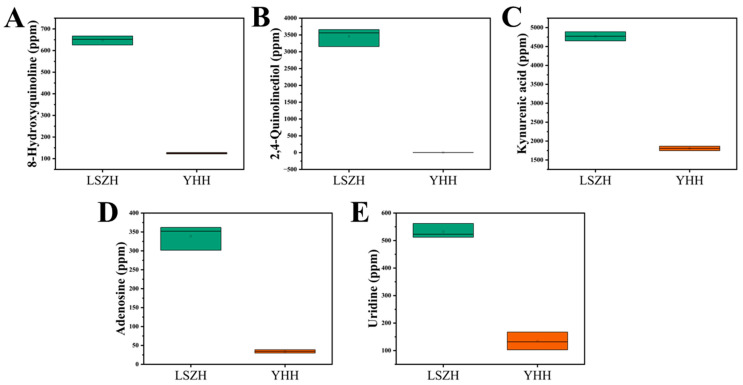
Quantitative results of compounds in LSZH and YHH. (**A**) 8-Hydroxyquinoline. (**B**) 2,4-Quinolinediol. (**C**) Kynurenic acid. (**D**) Adenosine. (**E**) Uridine.

To further confirm whether these bitter compounds are genuinely present in LSZH, we employed the UPLC-QQQ-MS/MS method to quantify the bitter compounds in LSZH and YHH. The results revealed that kynurenic acid, 8-hydroxyquinoline, adenosine, 2,4-quinolinediol, and uridine were accurately quantified in LSZH (Figure 9). The most abundant compound, kynurenic acid, reached a concentration of approximately 4500 ppm (Figure 9C). Moreover, the contents of these five compounds in YHH were extremely low compared to LSZH. Therefore, these five compounds are likely the primary sources of bitterness in LSZH.

In addition, Figure 8B presents the taste radar chart of the five bitter compounds identified in LSZH, and Figure 8C shows the normalized bitterness response values of these bitter compounds. It is clearly observable that the bitter response values of 8-hydroxyquinoline, adenosine, and uridine are higher than that of quinine, implying that they are more bitter than the standard bitter compound, quinine.

### 3.6. Molecular Docking Analysis

To further verify the potential mechanisms by which these compounds present a bitter taste, molecular docking was employed in this study to dock the bitter compounds with the bitter taste receptor TAS2R46, aiming to explore their interaction modes and strengths. The crystal structure of the human TAS2R46 receptor was resolved in 2022 (protein ID: 7XP4) and has been utilized in numerous molecular docking studies [16,36].

**Figure 10:**
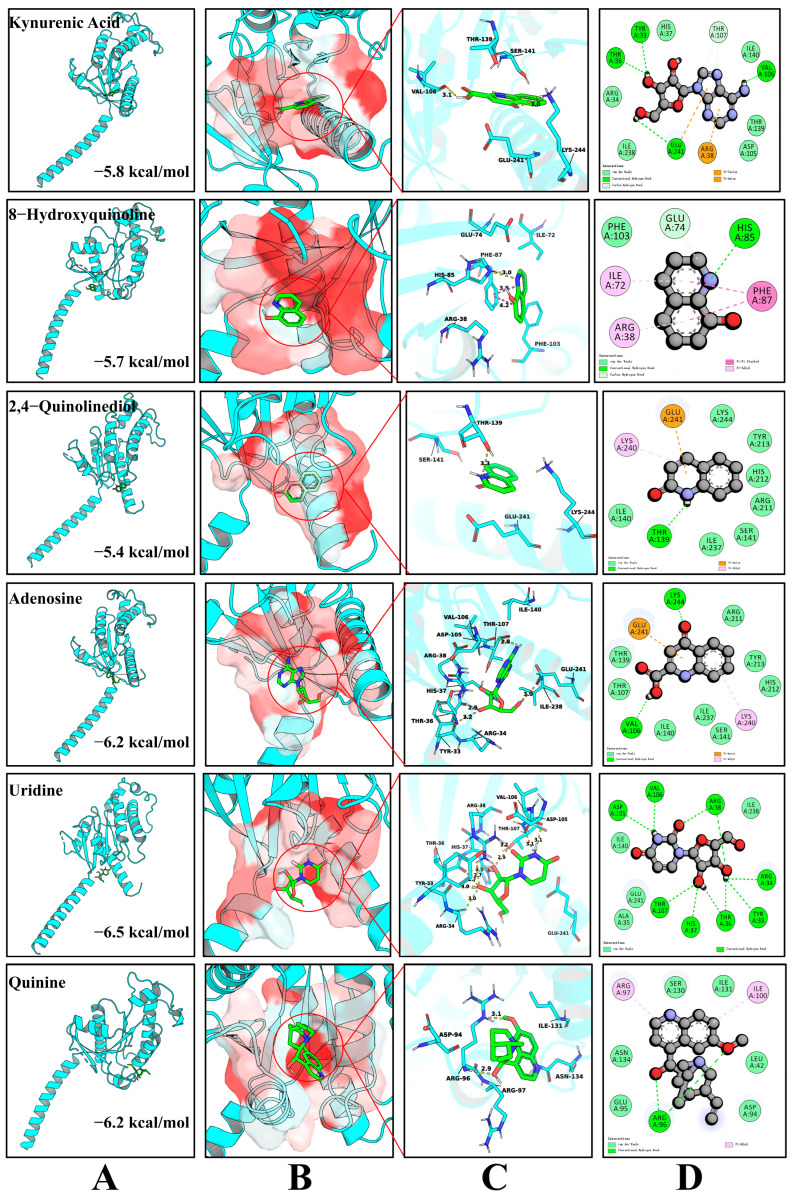
Molecular docking analysis of bitter compounds in LSZH with the bitter taste receptor. (**A**) Conformation of the complex formed by the bitter compound and the bitter taste receptor. (**B**) Bitter compound located in the hydrophobic pocket. (**C**) Three-dimensional interaction display. (**D**) Two-dimensional interaction display.

The molecular docking results demonstrated that the five bitter compounds—kynurenic acid, 8-hydroxyquinoline, adenosine, 2,4-quinolinediol, and uridine—could stably embed into the hydrophobic binding pocket of the bitter taste receptor. Their calculated binding affinities were kynurenic acid (−5.8 kcal/mol), 8-hydroxyquinoline (−5.7 kcal/mol), adenosine (−6.2 kcal/mol), 2,4-quinolinediol (−5.4 kcal/mol), and uridine (−6.5 kcal/mol), indicating strong affinities between these bitter compounds and TAS2R46. Among them, the docking binding affinities of adenosine (−6.2 kcal/mol) and uridine (−6.5 kcal/mol) with the bitter taste receptor were close to, or even lower than, that of quinine (−6.2 kcal/mol). As shown in Figure 10A, the bitter compounds are located in the center of the pocket formed by the multi-helical structure of the bitter taste receptor, surrounded by several α-helices. Figure 10B reveals that the binding pocket is predominantly composed of non-polar or weakly polar regions, suggesting that hydrophobic interactions play a leading role during the ligand binding process. The 3D interaction diagram (Figure 10C) and 2D diagram (Figure 10D) display the key interaction sites between the bitter compounds and the bitter taste receptor. The hydrogen bonding information from the molecular docking results is summarized in Table S2.

Taking adenosine and uridine as examples, adenosine formed four stable conventional hydrogen bonds with the GLU241, TYR33, THR36, and VAL106 residues of the bitter taste receptor. Concurrently, π-related interactions existed between uridine and residues such as GLU241 and ARG38. In addition, multiple residues, including HIS37 and ILE238, formed close contacts with adenosine through van der Waals forces and hydrophobic interactions, further enhancing the stability of the complex. Uridine formed 10 stable conventional hydrogen bonds with the ARG38, VAL106, ASP105, THR107, HIS37, THR36, TYR33, and ARG34 residues of the bitter taste receptor. Moreover, multiple residues such as GLU241 and ILE140 formed close contact with uridine through van der Waals forces and hydrophobic interactions, reinforcing the complex’s stability. Overall, hydrogen bonds, hydrophobic interactions, and π-related interactions collectively maintained the formation of stable complexes between the bitter compounds and the bitter taste receptor.

Generally, a binding affinity lower than −5 kcal/mol indicates a strong interaction between the ligand and the receptor [37]. The results of this study demonstrated that the five compounds identified in LSZH all possess the characteristics of bitter compounds. These findings indicate that the integrated approach employed in this study can successfully identify the characteristic bitter compounds in LSZH and elucidate the intrinsic reasons for its bitter taste, thereby providing a theoretical reference for the better development and utilization of LSZH.

## 4. Conclusions

Through a multidimensional analytical approach, this study systematically elucidated the chemical basis of bitterness in *Castanopsis fissa* honey and its interaction mechanisms with taste receptors. The results demonstrated that alkaloids constitute the core source of the bitter profile of LSZH in terms of both chemical diversity and abundance. We successfully constructed and applied a Random Forest machine learning model, which, combined with UPLC-QE-MS untargeted metabolomics, efficiently screened 71 candidate bitter compounds. Furthermore, five key taste markers (kynurenic acid, 8-hydroxyquinoline, adenosine, 2,4-quinolinediol, and uridine) were precisely identified and quantified via electronic tongue and targeted UPLC-QQQ-MS/MS analysis. Additionally, molecular docking analysis elucidated that these key bitter molecules stably bind to the hydrophobic pocket of the human bitter taste receptor TAS2R46, primarily through hydrogen bonding and hydrophobic interactions, thereby explaining the intrinsic cause of LSZH bitterness at the molecular level. This study not only provides direct chemical evidence for evaluating the unique flavor quality of *Castanopsis fissa* honey but also establishes a research paradigm for the high-throughput screening and mechanistic analysis of characteristic flavor compounds in complex food matrices.

## Data Availability

The original contributions presented in the study are included in the article/Supplementary Material, further inquiries can be directed to the corresponding authors.

## Supplementary Materials

The following supporting information can be downloaded at: https://www.mdpi.com/article/10.3390/foods15081379/s1. Table S1. Evaluation results of the machine learning models. Table S2. Statistical table of hydrogen bond information in molecular docking results.

## References

[1] Schiassi M.C.E.V., de Souza V.R., Lago A.M.T., Carvalho G.R., Curi P.N., Guimarães A.S., Queiroz F. (2021). Quality of honeys from different botanical origins. J. Food Sci. Technol..

[2] Yu W., Li X., Sun Q., Yi S., Zhang G., Chen L., Li Z., Li J., Luo L. (2024). Metabolomics and network pharmacology reveal the mechanism of Castanopsis honey against Streptococcus pyogenes. Food Chem..

[3] Xu Y., Sun Q., Chen W., Han Y., Gao Y., Ye J., Wang H., Gao L., Liu Y., Yang Y. (2022). The Taste-Masking Mechanism of Chitosan at the Molecular Level on Bitter Drugs of Alkaloids and Flavonoid Glycosides from Traditional Chinese Medicine. Molecules.

[4] Jiang L., Gong Y., Zhao Y., Dong W., Guo L., Ju J., Su N. (2024). Changes in Biochemical Composition and Nutrient Materials in Apocynum pictum Honey During Storage. Foods.

[5] Karolkowski A., Belloir C., Lucchi G., Martin C., Bouzidi E., Levavasseur L., Salles C., Briand L. (2023). Activation of bitter taste receptors by saponins and alkaloids identified in faba beans (*Vicia faba* L. minor). Food Chem..

[6] Huang J., Lu Y.-J., Guo C., Zuo S., Zhou J.-L., Wong W.-L., Huang B. (2021). The study of citrus-derived flavonoids as effective bitter taste inhibitors. J. Sci. Food Agric..

[7] Yang M., Mei Y., Lai H., Wang Y., Huang Y., Zeng X., Ge L., Zhao N. (2024). Characterization of the Universal Flavor in Chinese Butter Hotpot by Multiple Mass Spectrometry Detection Technology. J. Food Qual..

[8] Daher D., Deracinois B., Baniel A., Wattez E., Dantin J., Froidevaux R., Chollet S., Flahaut C. (2020). Principal Component Analysis from Mass Spectrometry Data Combined to a Sensory Evaluation as a Suitable Method for Assessing Bitterness of Enzymatic Hydrolysates Produced from Micellar Casein Proteins. Foods.

[9] Ziaikin E., David M., Uspenskaya S., Niv M.Y. (2025). BitterDB: 2024 update on bitter ligands and taste receptors. Nucleic Acids Res..

[10] Goel M., Grover N., Batra D., Garg N., Tuwani R., Sethupathy A., Bagler G. (2024). FlavorDB2: An updated database of flavor molecules. J. Food Sci..

[11] Margulis E., Dagan-Wiener A., Ives R.S., Jaffari S., Siems K., Niv M.Y. (2021). Intense bitterness of molecules: Machine learning for expediting drug discovery. Comput. Struct. Biotechnol. J..

[12] Tuwani R., Wadhwa S., Bagler G. (2019). BitterSweet: Building machine learning models for predicting the bitter and sweet taste of small molecules. Sci. Rep..

[13] Yu Y., Liu S., Zhang X., Yu W., Pei X., Liu L., Jin Y. (2024). Identification and prediction of milk-derived bitter taste peptides based on peptidomics technology and machine learning method. Food Chem..

[14] Jin X., Bi H., Jing X., Wang L., Lei J., Chai S., Yang X. (2024). Metabolomics and transcriptomics reveal metabolites and genes associated with the bitterness and astringency in sweet potato tips. Sci. Hortic..

[15] Bassoli A., Borgonovo G., Caremoli F., Mancuso G. (2014). The taste of D- and L-amino acids: In vitro binding assays with cloned human bitter (TAS2Rs) and sweet (TAS1R2/TAS1R3) receptors. Food Chem..

[16] Xu W., Wu L., Liu S., Liu X., Zhou C., Zhang J., Fu Y., Guo Y., Wu Y., Tan Q. (2022). Structural basis for strychnine activation of human bitter taste receptor TAS2R46. Science.

[17] Qiu H., Ma D., Dong B., Liu Z., Qian C. (2026). Identification of hTAS2R38 bitter taste receptor antagonists via homology modeling and molecular docking. Curr. Res. Food Sci..

[18] Li C., Yang D., Li L., Wang Y., Chen S., Zhao Y., Lin W. (2023). Comparison of the taste mechanisms of umami and bitter peptides from fermented mandarin fish (Chouguiyu) based on molecular docking and electronic tongue technology. Food Funct..

[19] (2012). Sensory Analysis–General Guidance for the Selection, Training, and Monitoring of Assessors, Part 1–Selected Assessors.

[20] Piana M.L., Cianciabella M., Daniele G.M., Badiani A., Rocculi P., Tappi S., Gatti E., Marcazzan G.L., Magli M., Medoro C. (2023). Influence of the Physical State of Two Monofloral Honeys on Sensory Properties and Consumer Satisfaction. Foods.

[21] Garg N., Sethupathy A., Tuwani R., Nk R., Dokania S., Iyer A., Gupta A., Agrawal S., Singh N., Shukla S. (2018). FlavorDB: A database of flavor molecules. Nucleic Acids Res..

[22] Jaeger S., Fulle S., Turk S. (2018). Mol2vec: Unsupervised Machine Learning Approach with Chemical Intuition. J. Chem. Inf. Model..

[23] Olaniran O.R., Alzahrani A.R.R., Alzahrani M.R. (2024). Eigenvalue Distributions in Random Confusion Matrices: Applications to Machine Learning Evaluation. Mathematics.

[24] Kim H.W., Wang M., Leber C.A., Nothias L.-F., Reher R., Kang K.B., van der Hooft J.J.J., Dorrestein P.C., Gerwick W.H., Cottrell G.W. (2021). NPClassifier: A Deep Neural Network-Based Structural Classification Tool for Natural Products. J. Nat. Prod..

[25] Hanwell M.D., Curtis D.E., Lonie D.C., Vandermeersch T., Zurek E., Hutchison G.R. (2012). Avogadro: An advanced semantic chemical editor, visualization, and analysis platform. J. Cheminformatics.

[26] Morris G.M., Huey R., Lindstrom W., Sanner M.F., Belew R.K., Goodsell D.S., Olson A.J. (2009). AutoDock4 and AutoDockTools4: Automated docking with selective receptor flexibility. J. Comput. Chem..

[27] Trott O., Olson A.J. (2010). AutoDock Vina: Improving the speed and accuracy of docking with a new scoring function, efficient optimization, and multithreading. J. Comput. Chem..

[28] Zhang Y.-F., Wang Y.-H., Gu Z.-F., Pan X.-R., Li J., Ding H., Zhang Y., Deng K.-J. (2023). Bitter-RF: A random forest machine model for recognizing bitter peptides. Front. Med..

[29] Banerjee P., Preissner R. (2018). BitterSweetForest: A Random Forest Based Binary Classifier to Predict Bitterness and Sweetness of Chemical Compounds. Front. Chem..

[30] Mahar R., Manivel N., Kanojiya S., Mishra D.K., Shukla S.K. (2022). Assessment of Tissue Specific Distribution and Seasonal Variation of Alkaloids in Alstonia scholaris. Metabolites.

[31] Estivi L., Buratti S., Fusi D., Benedetti S., Rodríguez G., Brandolini A., Hidalgo A. (2022). Alkaloid content and taste profile assessed by electronic tongue of Lupinus albus seeds debittered by different methods. J. Food Compos. Anal..

[32] Deng M., Hida N., Yamazaki T., Morishima R., Kato Y., Fujita Y., Nakamura A., Harada T. (2022). Comparison of Bitterness Intensity between Prednisolone and Quinine in a Human Sensory Test Indicated Individual Differences in Bitter-Taste Perception. Pharmaceutics.

[33] Kohl S., Behrens M., Dunkel A., Hofmann T., Meyerhof W. (2013). Amino Acids and Peptides Activate at Least Five Members of the Human Bitter Taste Receptor Family. J. Agric. Food Chem..

[34] Roland W.S.U., van Buren L., Gruppen H., Driesse M., Gouka R.J., Smit G., Vincken J.-P. (2013). Bitter Taste Receptor Activation by Flavonoids and Isoflavonoids: Modeled Structural Requirements for Activation of hTAS2R14 and hTAS2R39. J. Agric. Food Chem..

[35] Sonntag T., Kunert C., Dunkel A., Hofmann T. (2010). Sensory-Guided Identification of N-(1-Methyl-4-oxoimidazolidin-2-ylidene)-α-amino Acids as Contributors to the Thick-Sour and Mouth-Drying Orosensation of Stewed Beef Juice. J. Agric. Food Chem..

[36] Pedroni L., Perugino F., Kurtaga A., Galaverna G., Dall’Asta C., Dellafiora L. (2023). The bitter side of toxicity: A big data analysis spotted the interaction between trichothecenes and bitter receptors. Food Res. Int..

[37] Liu X., Wang M., Wu S., Wang H., Tie F., Hu N., Zhou W., Dong Q. (2026). Exploring the Anti-Inflammatory Molecular Mechanism of Gentiana szechenyii Kanitz. Based on UPLC–MS/MS Combined With Network Pharmacology, Molecular Docking, and Molecular Dynamics Simulation. Chem. Biodivers..

