# Supplementary material for: Elucidating the Material Basis and Receptor Mechanism of Bitterness in Castanopsis fissa Honey Using Machine Learning, Metabolomics, and Molecular Docking
Source: Foods. 2026 Apr 15;15(8):1379. doi: 10.3390/foods15081379 (PMC13114753; doi:10.3390/foods15081379)
Supplement: Supplementary file 1 [file foods-15-01379-s001.zip › foods-4204813-supplementary/Supplementary File/Supplementary File.pdf]

Supplementary File

Table S1. Evaluation results of the machine learning models.

	precision	recall	f1-score
**Xgboost**			
Bitter	0.81	0.82	0.81
Tasteless	0.7	0.85	0.77
Sweet	0.87	0.82	0.84
accuracy			0.82
macro avg	0.79	0.83	0.81
weighted avg	0.83	0.82	0.82
**LightGBM**			
	precision	recall	f1-score
Bitter	0.84	0.83	0.83
Tasteless	0.69	1	0.81
Sweet	0.9	0.83	0.87
accuracy			0.85
macro avg	0.81	0.89	0.84
weighted avg	0.86	0.85	0.85
**RandomForest**			
	precision	recall	f1-score
Bitter	0.83	0.84	0.83
Tasteless	0.73	1	0.85
Sweet	0.9	0.83	0.87
accuracy			0.85
macro avg	0.82	0.89	0.85
weighted avg	0.86	0.85	0.85
**MLP**			
	precision	recall	f1-score
Bitter	0.82	0.81	0.81
Tasteless	0.69	0.67	0.68
Sweet	0.82	0.83	0.83
accuracy			0.81
macro avg	0.79	0.77	0.77
weighted avg	0.81	0.81	0.81

Table S2. Statistical table of hydrogen bond information in molecular docking results.

Bitter compound	Binding affinity（kcal/mol）	Hydrogen bond number	Residue of amino acid involved
Kynurenic Acid	-5.8	2	LYS244, VAL106
8_Hydroxyquinoline	-5.7	1	HIS85
2,4-Quinolinediol	-5.4	1	THR139
Adenosine	-6.2	4	TYR33, THR36, VAL106, GLU241
Uridine	-6.5	10	VAL106, ASP105, ARG38, ARG34, TYR33, TYR36, HIS37, THR107
Quinine	-6.2	2	ARG96
